# The use of machine learning based models to predict the severity of community acquired pneumonia in hospitalised patients: A systematic review

**DOI:** 10.1177/17511437251315319

**Published:** 2025-02-03

**Authors:** Caitlin Lythgoe, David Oliver Hamilton, Brian W Johnston, Sandra Ortega-Martorell, Ivan Olier, Ingeborg Welters

**Affiliations:** 1 Department of Critical Care, Royal Liverpool University Hospital, Liverpool University Hospitals NHS Foundation Trust, Liverpool, UK; 2 Department of Cardiovascular and Metabolic Medicine, Institute of Life Course and Medical Sciences, University of Liverpool, Liverpool, UK; 3 Liverpool Centre for Cardiovascular Science at University of Liverpool, Liverpool John Moores University and Liverpool Heart & Chest Hospital, Liverpool, UK; 4 Data Science Research Centre, Liverpool John Moores University, Liverpool, UK

**Keywords:** Community acquired pneumonia, prediction model, critical care, intensive care, critically ill, machine learning

## Abstract

**Background:**

Community acquired pneumonia (CAP) is a common cause of hospital admission. CAP carries significant risk of adverse outcomes including organ dysfunction, intensive care unit (ICU) admission and death. Earlier admission to ICU for those with severe CAP is associated with better outcomes. Traditional prediction models are used in clinical practice to predict the severity of CAP. However, accuracy of predicting severity may be improved by using machine learning (ML) based models with added advantages of automation and speed. This systematic review evaluates the evidence base of ML-prediction tools in predicting CAP severity.

**Methods:**

MEDLINE, EMBASE and PubMed were systematically searched for studies that used ML-based models to predict mortality and/or ICU admission in CAP patients, where a performance metric was reported.

**Results:**

11 papers including a total of 351,365 CAP patients were included. All papers predicted severity and four predicted ICU admission. Most papers applied multiple ML algorithms to datasets and derived area under the receiver operator characteristic curve (AUROC) of 0.98 at best performance and 0.57 at worst, with a mixed performance against traditional prediction tools.

**Conclusion:**

Although ML models showed good performance at predicting CAP severity, the variables selected for inclusion in each model varied significantly which limited comparisons between models and there was a lack of reproducible data, limiting validity. Future research should focus on validating ML predication models in multiple cohorts to derive robust, reproducible performance measures, and to demonstrate a benefit in terms of patient outcomes and resource use.

## Introduction

Community-acquired pneumonia (CAP) represents a significant cause of morbidity, mortality, and health-care spending. In the United Kingdom alone, 29,000 deaths per year are attributed to CAP.^1^ Once patients are admitted to hospital, between 10% and 15% will be admitted to an Intensive Care Unit (ICU) and between 5% and 20% will die.^2,3^ There are 20,000 annual admissions to the ICU due to pneumonia, which represents 12% of all admissions,^4^ and higher mortality rates are seen in critically ill patients.^5^ The incidence of CAP is increasing globally, which has significant implications for resource use.^3^

The decision to admit a patient to the ICU is complex, taking into account the patient’s acuity of illness, chronic co-morbidities and wishes.^6^ Patients with severe CAP who are admitted to ICU earlier in their admission have better outcomes than those with similar severity who are admitted later.^5^ It is therefore essential to have accurate prediction tools to aid clinicians to identify patients who may benefit from earlier ICU admission.

Traditional severity prediction tools use a combination of clinical, radiological, microbiological, and laboratory results to predict CAP severity.^7^ Two frequently used tools are CURB-65 and the Pneumonia Severity Index (PSI). CURB-65, which is validated and endorsed by The British Thoracic Society (BTS), uses five variables^8^ and the PSI uses 20 variables to predict CAP severity.^9,10^ Previous systematic reviews have demonstrated that these scores have moderate to good accuracy in predicting mortality but are sub-optimal at predicting ICU admission.^2,11,12^ In ICU patients, mortality scores are often calculated at admission including APACHE II and Sequential Organ Failure Assessment (SOFA) score, which are used to predict mortality in patients with CAP.^13^ Traditional models can incur issues with adherence by clinical staff and practicability in a clinical environment.^14^

Machine learning (ML) is an increasingly popular method used to improve diagnostics and outcome prediction in medicine.^15^ ML involves the development of algorithms which automatically learn from existing data for their use in the prediction of future clinical events.^16^ Unlike conventional scoring systems that rely on predetermined rules and fixed parameters, ML algorithms can dynamically adapt and learn from vast datasets, capturing complex patterns and nuances in patient data. This adaptability allows ML models to continuously improve their predictive accuracy, identifying subtle correlations and individualised risk factors that might go unnoticed by traditional scoring tools. Additionally, ML can integrate diverse data sources, such as real-time patient monitoring, genetic information, and electronic health records (EHR), enabling a more comprehensive and personalised approach to risk stratification.

ML prediction models have the potential to be just as, if not more accurate than traditional prediction tools, and confer the advantages of automatic and prompt severity prediction.^15^ It is therefore important to systematically review the scope of ML studies in CAP severity prediction with an emphasis on mortality and escalation to critical care, and to identify areas of focus for future research. Since this is an area of rapid growth, an up-to-date search of the literature is necessary to include recent advances in the area and to aid clinical decision making for patients with CAP.^17^

## Aim

To review and evaluate the existing literature investigating machine learning based models used to predict severity in CAP.^18^

## Methods

This study was conducted in line with the PRISMA statement, a guideline for reporting systematic reviews.

## Study eligibility

We included research studies which reported derivation or validation of ML based risk prediction tools to predict the severity of CAP in patients >18 years old presenting to hospital with diagnosis of CAP. Despite the high number of COVID-19 related publications, these were omitted from this study as COVID-19 is a well-defined and distinct disease with different diagnostic and treatment pathways to CAP.

Inclusion criteria:

• All prospective and retrospective quantitative research studies that report derivation or validation of ML-based risk prediction tools used to predict severity of CAP
• Studies must describe at least one ML algorithm that is used to predict severity in CAP
• Studies must predict mortality and/or ICU admission
• Patients ⩾18 years only presenting to hospital with a diagnosis of CAP

Exclusion criteria:

• Studies of participants under 18 years, pregnant women, or immunocompromised patients
• Studies of participants with CAP limited to specific age categories, co-morbidities, or participant characteristics
• Studies reporting ML based tools for other specific types of pneumonia such as hospital acquired pneumonia, aspiration pneumonia or single organism pneumonia (e.g. COVID-19)
• Studies with no data related to the ML algorithm performance and/or performance was not separated from another model
• Qualitative studies, case studies, editorials, letters, abstract-only reports, reviews and commentaries that do not include original quantitative data

## Search methods

**Figure 1.**
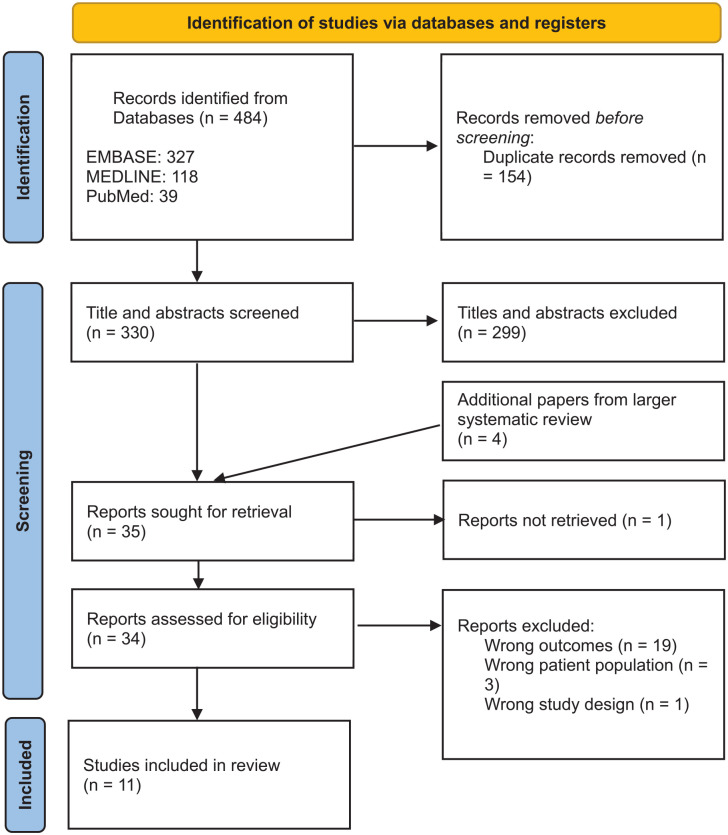
PRISMA diagram.

Electronic databases, EMBASE and MEDLINE, were searched for studies published up to June 2022. Due to the rapid growth of literature in this area, PubMed was also searched to include papers not yet added to MEDLINE. Search strategies were devised and conducted with specialist librarian input. The searches were performed again in May 2023 to include up to date papers. The results from the first and second search are combined in the PRISMA diagram (Figure 1).

## Study selection

Titles and abstracts were screened independently by two researchers (DOH and one of SG, BWJ), with any disagreements discussed and decided with a third researcher (IW). Full text screening was conducted by two researchers (SG, CL) with reasons given for exclusion, where there were discrepancies, this was discussed with a third researcher (IW). Papers were screened and selected for inclusion based on the inclusion and exclusion criteria.

## Data extraction

Data was extracted from the studies selected for inclusion and recorded in summary tables. Extracted data included study methodology, study population characteristics, ML algorithm, outcome measures and performance measures. In studies where there was both a derivation and validation cohort, the validation cohort’s results have been presented where possible. The area under the receiver operator characteristic curve (AUROC) is presented for all included studies, this predictive performance measure was chosen to present due to its ability to provide a nuanced and robust evaluation of predictive model discriminatory power across various thresholds, along with its wide use in the medical field, including previous use in the analysis of traditional predictive models for CAP.^11^ A descriptive summary of the main findings from the included papers is provided. The AUROC given for each study has been rounded to two decimal places for comparison between studies.

## Results

A total of 330 studies were identified from EMBASE, MEDLINE and PubMed searches. Following title and abstract screening, the full texts of 35 studies were retrieved and assessed for eligibility resulting in 11 studies being included in this review (Figure 1).^17,19–28^

Most of the included studies were published in the last 4 years with three studies published before 2019. Data from a total of 351,365 patients was used for model development in the included papers, with sample sizes ranging from 1210 to 297,498. All studies included both training (derivation) and test (evaluation) sets of patients, although notably only one study included an external validation group.^21^ Seven studies utilised existing datasets including the PROGRESS study,^29^ PORT database,^30^ GenIMS study database,^31^ Cerner database^32^ and Veterans Affairs network database.^33^The remaining studies utilised hospital medical records for data and only one study validated their model using a prospective cohort.^17^ Table 1 describes the studies in more detail.

**Table 1.** A summary of the studies included in this review.

Study	CAP definition	Data source	Study design	Setting (no. centres)	Sample size	Derivation/evaluation	Validation
Visweswaran^20^^a^ Cooper^19^^a^ Tajgardoon^22^^a^	One or more symptoms suggestive of pneumonia and radiological evidence of pneumonia	PORT database (30)	Retrospective analysis of prospective cohort	Multicentre study: US (4), Canada (1)	2287	Both	Internal validation (cross validation)
					Training: 1601		
					Test set: 686		
Wu^21^	Clinical and radiologic diagnosis of pneumonia as per ICD-9-CM	GenIMS study database (31)	Retrospective analysis of prospective cohort	Multicentre study: US (28)	1815	Both	External validation
Przybilla^23^	NR	PROGRESS study (29)	Retrospective analysis of observational study data	Multicentre study: Germany (58), Austria (2)	2005	Both	Internal validation (subset of severe CAP cases)
					Training: 1863		
					Test set: 142		
Feng^24^	Presence of a new pulmonary infiltrate associated with at least one predefined clinical/microbiological/biochemical criterion	Hospital medical record	Retrospective case control	Single centre, China	3997	Both	Internal validation
Wang^34^	ICD-9 and ICD-10 diagnosis codes of CAP	Cerner database (32)	Retrospective observational	Multi-centre, US (749)	34,720	Both	Internal validation
					Training: 80%		

Test set: 20%	Quah^17^	Physician-determined diagnosis of CAP at emergency department presentation	Electronic medical records	Derivation: retrospective cohort validation: prospective cohort	Single centre, Singapore	Training: 1966	Both
Temporal validation						Test set: 302	
	Jones^26^	Diagnostic codes assigned in ED/ clinical diagnosis of pneumonia within the ED physician document (recognised by natural language processing)	Veterans affairs network database	Retrospective analysis of cohort	Multi-centre, US (117)	297,498	Both
Temporal validation						Training: 230,470	
						Test set: 67,028	
	Yuan^27^	According to Chinese Guidelines for Diagnosis and Treatment of Adult Community-acquired Pneumonia	Hospital records	Retrospective case-control	Single centre hospital, China	1210	Both
Internal validation						Training: 726	
						Test set: 484	
	Cilloniz^28^	Presence of new acute respiratory symptoms, signs, and compatible infiltrate(s) on chest radiographs	Hospital records	Retrospective cohort study	Multi-centre, Spain (2)	5565	Both
External validation						Training: 4531	
						Test set: 1034	

CAP: community acquired pneumonia; NR: not reported.

a Denotes paired papers.

In terms of geographical distribution, six studies included data from the US, three from Canada, two from China and one from each of: Singapore, Spain, Germany and Austria.

One research group produced three papers included in this review using the same dataset.^19,20,22^

The patient data was used to inform the variables in ML algorithms. Table 2 denotes the types of variables included in the ML models in each paper.

**Table 2.** Variables included in ML Algorithms in each included study.

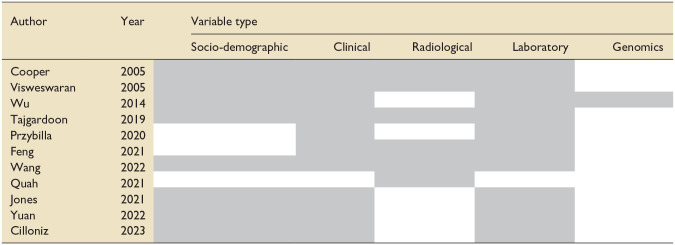

Shaded boxes highlights the category of variables that were included in machine learning algorithms in each individual study.

The most common types of ML algorithms used were neural networks (seven), forms of Naïve Bayesian Classifiers (six), random forest (five) and support vector machine (four). Logistic regression (a statistical method) was used alongside ML algorithms in eight studies and these results have been included for comparison. The number of variables used in the algorithms varied from one^17^ to 196 variables.^20^

All studies designed models to predict mortality or a pre-specified dire outcome which included mortality or ICU admission. The highest reported AUROC predicted by a ML model was 0.98,^24^ the lowest 0.57.^34^ Of the 59 ML models described in the studies; most had an AUROC between 0.80 and 0.90 (Table 3).

**Table 3.** Area under receiver operating characteristic curve (AUROC) for each machine learning (ML) algorithm in the included papers.

Study	Outcomes reported	ML algorithm	AUROC	CI	Number of variables/predictors	Comparison to traditional models
Cooper^19^	‘Dire Outcome’ described as one of:	FAN.C	0.81^a^	NR	161	Not performed
	[1] 30-day mortality					
	[2] ICU admission for respiratory failure, respiratory/cardiac arrest, or shock					
	[3] the presence of one or more defined severe complications	FAN.D	0.85^a^	NR	158	
		FMM.C	0.82^a^	NR	161	
		FMM.D	0.81^a^	NR	158	
		LR.DIRE	0.77^a^	NR	102	
		**NN.MTLR**	**0.86** ^a^	**NR**	**196**	
		NN.STL	0.85^a^	NR	196	
		RL.BS	0.85^a^	NR	22	
		SB.C	0.85^a^	NR	161	
		SB.D	0.85^a^	NR	158	
		SB.VS.D	0.81^a^	NR	46	
Visweswaran^20^a	‘Dire Outcome’ described as one of:	LR	0.74	(0.68–0.80)	196	Not performed
	[1] 30-day mortality					
	[2] ICU admission for respiratory failure, respiratory/cardiac arrest, or shock	ANN	0.83	(0.78–0.87)	196	
	[3] the presence of one or more defined severe complications	KNN	0.79	(0.74–0.84)	196	
		SB	0.85	(0.82–0.88)	196	
		**Modified LBR**	**0.86**	**(0.83**–**0.90)**	**196**	
		PSA	0.85	(0.82–0.88)	196	
Wu^21^	90-day mortality	NB	0.75	NR	15	Not performed
		SVM	0.75	NR	15	
		NN	0.76	NR	15	
		LR	0.74	NR	15	
		BL	0.75	NR	15	
		RT	0.66	NR	15	
		RF	0.70	NR	15	
		**DNF**	**0.79**	**NR**	**15**	
Tajgardoon^22^	‘Dire Outcome’ described as one of:	LR-L1	0.84^a^	(0.81–0.87)	41	Not performed
	[1] 30-day mortality					
	[2] ICU admission for respiratory failure, respiratory/cardiac arrest, or shock	LR-L2	0.84^a^	(0.81–0.87)	41	
	[3] the presence of one or more defined severe complications	NB	0.84^a^	(0.81–0.87)	41	
		SVM	0.84^a^	(0.81–0.87)	41	
		**RF**	**0.85** ^a^	**(0.82**–**0.88)**	**41**	
Przybilla^23^	28-day mortality	Markov disease states	0.76^a^	(0.70–0.83)	6 (1 data point)	PSI: 0.78 (0.71–0.86)
		**Markov model**	**0.89** ^a^	**(0.84**–**0.94)**	**6 (6 data points)**	CURB-65: 0.84 (0.76–0.89)
Feng^24^	Poor outcome defined as ‘death during hospitalisation’	**FCNN**	**0.98**	**NR**	**62**	FCNN model (derived model) compared to other machine learning models
		LR	0.80	NR	62	
		SVM	0.84	NR	62	
		KNN	0.78	NR	62	
		GNB	0.81	NR	62	
		DT	0.84	NR	62	
		RF	0.82	NR	62	
Wang^34^	In-hospital mortality	**RF**	**0.81**	**NR**	**20**	PSI: 0.77
		MLP	0.80	NR	20	
		LR	0.80	NR	20	
		LDA	0.79	NR	20	
		GNB	0.77	NR	20	
		SGD	0.75	NR	20	
		SVM	0.73	NR	20	
		KNN	0.69	NR	20	
		DT	0.57	NR	20	
Quah^17^	30-day mortality	CNN (CAPE)	0.79	(0.73–0.85)	1	CURB-65: 0.76 (0.70–0.81)
						PSI: 0.80 (0.74–0.86)
						**CAPE** **+** **PSI: 0.84 (0.79**–**0.89)**
						CAPE **+** CURB65: 0.83 (0.77–0.88)
Jones^26^	30-day morality	LR PSI	0.80	(0.79–0.80)	20	ePSI: 0.77 (0.77–0.78)
		XGB	0.84	(0.83–0.85)	19	18 variables
		XGB 28 variable	0.87	(0.86–0.87)	28	
		**XGB 69 variable**	**0.88**	**(0.87–0.88)**	**69**	
Yuan^27^	28-day mortality	**RF**	**0.96**	**(0.94**–**0.99)**	**106**	Not performed
		XGB	0.94	(0.90–0.98)	106	
		DNN	0.91	(0.86–0.96)	106	
		CNN	0.94	(0.91–0.97)	106	
		LR	0.71	(0.65–0.78)	106	
Cilloniz^28^	30-day mortality	CPN	0.83	(0.75–0.90)	22	**PSI: 0.83 (0.75**–**0.90)**
						SOFA: 0.73 (0.59–0.87)
						CURB-65: 0.76 (0.69–0.83)
						SOFA-imputed: 0.77 (0.71–0.84)
						qSOFA: 0.73 (0.65–0.80)

ANN/NN: (Artificial) Neural Network; BL: Boosted Logistic Regression; CAPE: Community Acquired; Pneumonia Artificial Intelligence Predictive Engine; CNN: Convolutional Neural Network; CPN: Causal Probabilistic Network; DNF: Disjunctive Normal Form; DNN: Deep Neural Network; DT: Decision Tree; FAN.C: Finite Mixture Segmented Naïve Bayes (Continuous); FAN.D: Finite Mixture Segmented Naïve Bayes (Discrete); FCNN: Fully Connected Neural Network; FMM.C: Finite Mixture Model (Continuous); FMM.D: Finite Mixture Model (Discrete); KNN: *K*-Nearest Neighbour; LR: Logistic Regression; LR.DIRE: Logistic Regression To Predict Dire Outcomes; LR-L1: Logistic Regression With L1 Regularisation; LR-L2: Logistic Regression With L2 Regularisation; MLP: Multi-Layer Perception; Modified LBR: Lazy Bayesian Rule; NB: Naïve Bayes; NN.MTLR: Neural Network Multitask Learning; NN.STL: Standard Neural Network Method; PSA: Patient Specific Algorithm; PSI: Pneumonia Severity Index; RF: Random Forest; RL.BS: Rule Based Learning With Bias Search; RT: Random Tree; SB: Simple Bayes; SB.C: Simple Bayes (Continuous); SB.D: Simple Bayes (Discrete); SB.VS.D: Simple Bayes Model Using Variable Selection (Discrete); SGD: Stochastic Gradient Descent; SVM: Support Vector Machine; XGB: Extreme Gradient Boosting (Boosted Decision Tree Algorithm).

a Indicates AUROC is from derivation cohort.

Five papers compared performance of their ML models to an existing prediction tool, with four comparing against PSI^17,23,28,34^, three comparing against CURB-65^17,23,28^, one comparing against a modified PSI (ePSI)^26^ and one comparing against a SOFA and modified SOFA score.^28^ The AUROC was higher for the ML model than the traditional model in three of the five papers.^17,23,26^

## Risk of bias

The PROBAST tool, a tool used to assess the risk of bias (ROB) and applicability of prediction model studies, was used to examine the risk of bias of the studies included in this review.^35^ Six of the 11 papers included in this review were judged to have a high risk of bias (Table 4). Almost all studies selected participants appropriately. For three papers, the outcome measure was determined as being too broad with high risk of co-founding factors and the use of predictors in the outcome definition.^19,20,22^ Multiple papers had issues with a lack of information surrounding handling of missing data, continuous and categorical predictors, censoring and sampling of control participants. External validation is a vital part of prediction model development and is often underperformed in development studies.^36^ Of note, only two papers used external validation to validate the ML prediction tool.^21,28^ Other studies attempted to offset this by using temporal validation, which carries the limitations of a random split sample approach.^17,26^

**Table 4.** PROBAST risk of bias.

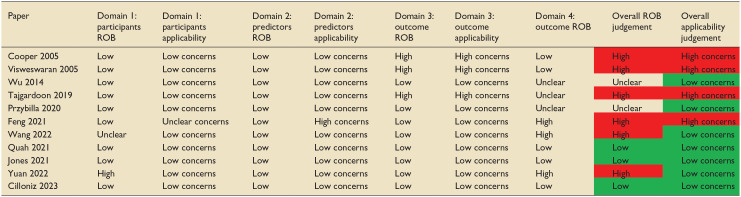

## Discussion

### Study characteristics

This review represents an up-to-date assessment of the application and accuracy of ML-based prediction models to predict severity in CAP. We identified 11 studies that utilised ML models to predict either mortality or dire outcome. Patients hospitalised with CAP are at risk of adverse outcomes including ICU admission and death. Given the prevalence of CAP and its associated mortality, it is important that we can accurately predict these adverse outcomes to aid decision making and resource allocation.

It is acknowledged that due to the high volume of patients presenting to hospital with CAP even small differences in the accuracy of CAP prediction scores can make a significant difference in terms of decision making and resource allocation.^11^

The majority of studies in this review were published from 2019 onwards, which may represent the increasing use of ML in recent years. During the COVID-19 pandemic, ML-based identification of risk factors and development of predictive scores were utilised to support decision making in healthcare systems under pressure.^37^ Indeed, one of the identified advantages of ML is the ability to easily change parameters based on local environmental factors, such as scarce resources as seen during a pandemic.

Many of the ML models in our review had a higher number of variables than traditional prediction models, with some ML models using 196 variables. This presents obvious barriers to clinical use, with the balance being between accuracy of the ML model versus practicality of its use in clinical practice. Electronic health records (EHR) may help mitigate this, but this relies on data being inputted and the integration of a model into the EHR.

### Clinical use of ML based prediction models

Machine learning models have the potential to aid clinicians in their decision making, supporting data driven communication between clinical teams and with patients, however, there are several barriers to their use which are important to consider in the design process. One of the main criticisms of ML models and their integration in clinical practice is the ‘black box’ nature of their prediction.^38^ Many advanced ML algorithms, such as deep neural networks, operate as complex, non-linear systems with numerous parameters, making it challenging to interpret how the model arrives at a specific prediction. This opacity in the decision making and justification for the outcome can make ML models difficult to interpret clinically and are often a barrier to their use. A previous systematic review found that although healthcare professionals perceived ML based prediction tools as adding value to decision making, barriers to their use include concerns regarding the quality of the data used to build tools, how data is used to produce predictions, and a lack of transparency with this process.^39^ The same study highlights the importance of explanations for model outcomes. We are recently witnessing the development of more interpretable ML models and techniques to provide explanations for the decisions made by complex ML algorithms.^40^ One of the papers in this review tries to mitigate for this by creating a model using Local Interpretable Model-Agnostic Explanations (LIME) to provide clinicians with explanations for the variables used in the ML model.^22^

### Performance of ML based models

Most studies described the performance of multiple ML algorithms, demonstrating both the breadth of ML models and the ease of applying multiple models to large cohorts of data. AUROC was used as an indication of model performance, generally it is accepted that AUROC 0.70–0.80 is considered acceptable, 0.80–0.90 is considered excellent and more than 0.90 is considered outstanding.^41^ The performance of ML-based models varied from below acceptable to outstanding, with most being excellent. There was variation between the performance of different ML models when applied to each study and some studies demonstrated high or low AUROCs irrespective of the ML algorithm.^21,24,27,34^ This may be indicative of the model performance being largely informed by the quality of the data input and selected variables which is supported by the similar AUROC for the three studies in the review which utilised the same dataset, despite using different ML models.^19,20,22^

All studies described both derivation and internal validation of ML algorithms however only one study described external validation of a model. This is important when interpreting the performance of the ML models as external validation is the gold standard for assessing the performance of ML prediction models, and is vital to assess applicability to medical practice and generalisability.^42^ Although there is a focus on using multiple models employing different ML algorithms, studies reproducing or validating these models in different patient cohorts are lacking.

### Validation of ML-based models

Future research must address the limitations highlighted in the current literature by focussing on robust external validation of ML models predicting the severity of CAP. External validation, recognised as the gold standard for assessing model performance, is essential to determine generalisability across diverse patient populations and healthcare settings.^43^ Multi-centre studies using datasets from varied geographical, demographic, and clinical contexts will be critical to achieve this.

Another key area for validation is the prospective evaluation of ML models in real-world clinical workflows. While retrospective datasets are instrumental in model derivation, prospective studies can help assess performance in dynamic clinical environments. Embedding these models in hospital settings can help gauge their real-time predictive accuracy, clinician acceptance, and operational impact on patient outcomes. Such studies can also provide evidence on how ML tools affect clinical resource allocation, such as guiding decisions on intensive care admissions or early discharge.

Furthermore, the role of explainable AI (XAI) will be integral to validation efforts, ensuring that predictions made by ML models can be understood and trusted by clinicians.^44,45^ Explainability can improve the interpretability of model outputs, enabling healthcare professionals to evaluate predictions in the context of established clinical reasoning. To enhance adoption, future studies should incorporate clinician input during model development and validation phases, aligning model outputs with clinical needs and workflows. Ultimately, these steps will strengthen the credibility of ML-based prediction tools, paving the way for their safe and effective implementation in routine care for CAP patients.

### Comparing ML based models and traditional models

In two of the five studies which compared a traditional method to an ML algorithm, the traditional method demonstrated a higher AUROC. A previous review showed an AUROC for CURB-65, PSI, and SOFA scores of 0.79, 0.82 and 0.78, respectively.^11,13^ These results show that there is acceptable to excellent performance of these traditional models at predicting severity in CAP, and they have the additional benefit of being highly researched and validated in multiple patient populations.^11^ Traditional models are easily accessible and interpretable for clinicians and can often be performed at the bedside with minimal resource use. For ML algorithms to be beneficial in clinical practice, their performance against traditional models would have to be demonstrably superior. Interestingly, in our review, some studies used traditional model scores as variables within the ML algorithm which made it difficult to interpret the performance of these ML models in comparison with traditional models.

One of the studies in this review combined the use of a machine learning model with traditional prediction models.^17^ Integrating machine learning into an already validated model (PSI and CURB-65) improved the performance of the traditional model. Integration of ML and traditional statistical prediction models has been used in other fields and found to provide more accurate and generalisable models for disease risk prediction then using each method alone. The synergistic use of ML and traditional models also has the potential to improve prediction accuracy whilst maintaining end-user understanding and interpretation of the outcome.

### Future outcome research and machine learning

As EHR based systems become commonplace integration of ML models into existing systems will increase, with the benefit of utilising and learning from local data and dynamically adapting to local changes. This will be particularly beneficial in patients with CAP given the association with locally confined population characteristics and the possibility of geographically diverse variants. Additionally, the ability to adjust thresholds based on population needs and resources is likely to improve clinical applicability.

One of the advantages of ML is the ability to detect complex, non-linear relationships between variables and outcomes.^46^ This makes ML more suited to ‘real world’ problem solving. Linking data from EHR’s with ML models allows capture of temporal relationships to detect disease earlier, as has been demonstrated for a diagnosis of heart failure.^47^ Analysis of such high granularity temporal data means that ML models can predict important additional outcomes such as length of stay, ICU readmission and complications, all of which are clinically important and relevant for healthcare planning and cost analysis.^48^

However, future research needs to focus on demonstrating not only the superiority of ML models, but also their ability to improve quality in terms of patient outcomes, impact on resource use and cost effectiveness. Until this point, perhaps the use of ML to support traditional models as is demonstrated in some of the studies in our review may be the first step towards integrating them in clinical practice.^17^

### The potential of machine learning

ML techniques have the potential to revolutionise how clinicians work in the future. In recent years there has been a rapid expansion of ML techniques in the published literature and ML is being utilised in many areas including, sepsis prediction,^49^ mortality and length of stay forecasting,^50^ image analysis,^51^ drug dosing optimisation,^52^ ventilator management^51^ and resource utilisation.^50^ The authors are unaware of any machine learning models that are currently in day-to-day use or implemented within ICU workflows. If the benefit of ML is to be realised, then embedding ML techniques in daily ICU practice is required.

An exciting avenue is the development of clinician support tools through the use of digital twins.^53^ Virtual twin-based models integrate multiple sources of information including, disease risk factors, comorbidities, imaging and biomarkers with the aim of bridging the gap between research and clinical practice by creating digital representations of individual patients. These personalised digital models and the use of in-silico modelling may 1 day allow real time predictive decision making to improve patient care.^54^

## Conclusion

This systematic review describes the current evidence base for ML prediction tools in predicting the severity of CAP in terms of ICU admission and mortality. Whilst the accuracy of several algorithms is excellent, there is substantial variation in the literature between the ML techniques used, the quality of these tools, and their accuracy in predicting mortality. This – alongside issues with the implementation and interpretation of ML prediction tools– means that currently there is insufficient evidence to regard these tools as superior to traditional measures for predicting CAP severity and guiding clinical decision making for patients with CAP. Further research is required, focussing on validating ML prediction tools, improving clinical interpretation of ML models, and demonstrating their ability to improve patient outcomes, impact on resource use and cost effectiveness.

### Recommendations for future research

1. Future studies should focus on creating robust and externally validated machine learning prediction tools for CAP severity prediction.
2. Studies describing the development of a CAP severity prediction tool should include clear information regarding handling of complexities in the data in order to minimise risk of bias.
3. There should be further research on interpretability of machine learning based prediction tools and the impact on clinical practice.
4. Future studies should focus on demonstrating the ability of machine learning based prediction tools to positively impact resource use and be cost effective.
5. Future studies should focus on demonstrating the ability of machine learning based prediction tools to improve patient outcomes.

## Supplemental Material

sj-docx-1-inc-10.1177_17511437251315319 – Supplemental material for The use of machine learning based models to predict the severity of community acquired pneumonia in hospitalised patients: A systematic review Supplemental material, sj-docx-1-inc-10.1177_17511437251315319 for The use of machine learning based models to predict the severity of community acquired pneumonia in hospitalised patients: A systematic review by Caitlin Lythgoe, David Oliver Hamilton, Brian W Johnston, Sandra Ortega-Martorell, Ivan Olier and Ingeborg Welters in Journal of the Intensive Care Society
