# Supplementary material for: The use of machine learning based models to predict the severity of community acquired pneumonia in hospitalised patients: A systematic review
Source: J Intensive Care Soc. 2025 Feb 3;26(2):237–48. doi: 10.1177/17511437251315319 (PMC11791961; doi:10.1177/17511437251315319)
Supplement: sj-docx-1-inc-10.1177_17511437251315319 – Supplemental material for The use of machine learning based models to predict the severity of community acquired pneumonia in hospitalised patients: A systematic review [file sj-docx-1-inc-10.1177_17511437251315319.docx]

**Supplementary Material**

**The Use of Machine Learning Based Models to Predict the Severity of Community Acquired Pneumonia in Hospitalised Patients: A Systematic Review**

**Authors:** Caitlin Lythgoe¹, David Oliver Hamilton¹, Brian W Johnston^1,2^*, Sandra Ortega-Martorell^3,4^, Ivan Olier^3,4^, Ingeborg Welters^1,2^

Full Electronic Search Embase and Medline

Embase

| 1 | ("community acquired" adj2 pneumonia).ab,ti. | 17,184 |
| --- | --- | --- |
| 2 | (communityacquired adj2 pneumonia).ab,ti. | 327 |
| 3 | exp community acquired pneumonia/ | 17,900 |
| 4 | 1 or 2 or 3 | 23,554 |
| 5 | "clinical decision rule*".ab,ti. | 1,402 |
| 6 | "clinical prediction rule*".ab,ti. | 1,654 |
| 7 | "clinical decision score*".ab,ti. | 21 |
| 8 | "clinical prediction score*".ab,ti. | 270 |
| 9 | (mortality adj4 predict*).ab,ti. | 86,897 |
| 10 | (outcome* adj4 predict*).ab,ti. | 188,121 |
| 11 | (risk* adj2 predict*).ab,ti. | 75,416 |
| 12 | (risk* adj2 stratif*).ab,ti. | 81,355 |
| 13 | (risk* adj2 calculat*).ab,ti. | 19,300 |
| 14 | (mortality adj4 risk*).ab,ti. | 130,876 |
| 15 | "curb 65".ab,ti. | 1,192 |
| 16 | curb65.ab,ti. | 266 |
| 17 | "curb criteria".ab,ti. | 1 |
| 18 | (PSI adj2 score*).ab,ti. | 769 |
| 19 | "pneumonia severity index".ab,ti. | 1,109 |
| 20 | "decision tree*".ab,ti. | 16,905 |
| 21 | (severity adj2 assess*).ab,ti. | 26,544 |
| 22 | (ICU adj2 admission*).ab,ti. | 29,391 |
| 23 | (ICU adj2 admit*).ab,ti. | 6,951 |
| 24 | (ICU adj2 need*).ab,ti. | 2,438 |
| 25 | ("intensive care" adj2 admission*).ab,ti. | 12,865 |
| 26 | (risk* adj2 prognostic*).ab,ti. | 6,804 |
| 27 | (prognostic* adj2 tool*).ab,ti. | 8,845 |
| 28 | "prediction model*".ab,ti. | 37,361 |
| 29 | exp clinical decision rule/ | 403 |
| 30 | exp CURB-65 score/ | 311 |
| 31 | exp Pneumonia Severity Index/ | 1,170 |
| 32 | 5 or 6 or 7 or 8 or 9 or 10 or 11 or 12 or 13 or 14 or 15 or 16 or 17 or 18 or 19 or 20 or 21 or 22 or 23 or 24 or 25 or 26 or 27 or 28 or 29 or 30 or 31 | 625,135 |
| 33 | 4 and 32 | 3,930 |
| 34 | limit 33 to (adult <18 to 64 years> or aged <65+ years>) | 2,526 |

Medline

| 1 | ("community acquired" adj2 pneumonia).ab,ti. | 11,493 |
| --- | --- | --- |
| 2 | (communityacquired adj2 pneumonia).ab,ti. | 15 |
| 3 | "community acquired pneumonia".ab,ti. | 11,012 |
| 4 | 1 or 2 or 3 | 11,499 |
| 5 | "clinical decision rule*".ab,ti. | 921 |
| 6 | "clinical prediction rule*".ab,ti. | 1,191 |
| 7 | "clinical decision score*".ab,ti. | 4 |
| 8 | "clinical prediction score*".ab,ti. | 162 |
| 9 | (mortality adj4 predict*).ab,ti. | 52,398 |
| 10 | (outcome* adj4 predict*).ab,ti. | 120,442 |
| 11 | (risk* adj2 predict*).ab,ti. | 48,929 |
| 12 | (risk* adj2 stratif*).ab,ti. | 46,286 |
| 13 | (risk* adj2 calculat*).ab,ti. | 12,206 |
| 14 | (mortality adj4 risk*).ab,ti. | 86,769 |
| 15 | "curb 65".ab,ti. | 660 |
| 16 | curb65.ab,ti. | 105 |
| 17 | "curb criteria".ab,ti. | 1 |
| 18 | (PSI adj2 score*).ab,ti. | 420 |
| 19 | "pneumonia severity index".ab,ti. | 757 |
| 20 | "decision tree*".ab,ti. | 11,943 |
| 21 | (severity adj2 assess*).ab,ti. | 16,248 |
| 22 | (ICU adj2 admission*).ab,ti. | 14,141 |
| 23 | (ICU adj2 admit*).ab,ti. | 2,481 |
| 24 | (ICU adj2 need*).ab,ti. | 1,143 |
| 25 | ("intensive care" adj2 admission*).ab,ti. | 9,135 |
| 26 | (risk* adj2 prognostic*).ab,ti. | 4,293 |
| 27 | (prognostic* adj2 tool*).ab,ti. | 5,325 |
| 28 | "prediction model*".ab,ti. | 27,708 |
| 29 | exp clinical decision rule/ | 868 |
| 30 | ("CURB-65" adj3 score*).ab,ti. | 395 |
| 31 | "psi score*".ab,ti. | 292 |
| 32 | 5 or 6 or 7 or 8 or 9 or 10 or 11 or 12 or 13 or 14 or 15 or 16 or 17 or 18 or 19 or 20 or 21 or 22 or 23 or 24 or 25 or 26 or 27 or 28 or 29 or 30 or 31 | 398,119 |
| 33 | 4 and 32 | 2,095 |
| 34 | limit 33 to "all adult (19 plus years)" | 1,410 |
